# Diminished Feedback Evaluation and Knowledge Updating Underlying Age-Related Differences in Choice Behavior During Feedback Learning

**DOI:** 10.3389/fnhum.2021.635996

**Published:** 2021-03-05

**Authors:** Tineke de Haan, Berry van den Berg, Marty G. Woldorff, André Aleman, Monicque M. Lorist

**Affiliations:** ^1^Department of Experimental Psychology, University of Groningen, Groningen, Netherlands; ^2^Center for Cognitive Neuroscience, Duke University, Durham, NC, United States; ^3^Department of Biomedical Sciences of Cells and Systems, University Medical Center Groningen, University of Groningen, Groningen, Netherlands

**Keywords:** aging, fMRI, probabilistic learning, feedback, choice behavior

## Abstract

In our daily lives, we continuously evaluate feedback information, update our knowledge, and adapt our behavior in order to reach desired goals. This ability to learn from feedback information, however, declines with age. Previous research has indicated that certain higher-level learning processes, such as feedback evaluation, integration of feedback information, and updating of knowledge, seem to be affected by age, and recent studies have shown how the adaption of choice behavior following feedback can differ with age. The neural mechanisms underlying this age-related change in choice behavior during learning, however, remain unclear. The aim of this study is therefore to investigate the relation between learning-related neural processes and choice behavior during feedback learning in two age groups. Behavioral and fMRI data were collected, while a group of young (age 18–30) and older (age 60–75) adults performed a probabilistic learning task consisting of 10 blocks of 20 trials each. On each trial, the participants chose between a house and a face, after which they received visual feedback (loss vs. gain). In each block, either the house or the face image had a higher probability of yielding a reward (62.5 vs. 37.5%). Participants were instructed to try to maximize their gains. Our results showed that less successful learning in older adults, as indicated by a lower learning rate, corresponded with a higher tendency to switch to the other stimulus option, and with a reduced adaptation of this switch choice behavior following positive feedback. At the neural level, activation following positive and negative feedback was found to be less distinctive in the older adults, due to a smaller feedback-evaluation response to positive feedback in this group. Furthermore, whereas young adults displayed increased levels of knowledge updating prior to adapting choice behavior, we did not find this effect in older adults. Together, our results suggest that diminished learning performance with age corresponds with diminished evaluation of positive feedback and reduced knowledge updating related to changes in choice behavior, indicating how such differences in feedback processing at the trial level in older adults might lead to reduced learning performance across trials.

## Introduction

In order to successfully interact with our environment and reach desired goals, we continuously learn from the outcomes of our actions and choices. These outcomes (feedback information) are evaluated in the light of the goals we aim to achieve and are integrated with the outcomes from prior experiences. In addition, based on choice-outcome contingencies, knowledge will be updated, enabling future behavior to be adapted accordingly. Although learning is crucial at all ages, research has shown that the ability to learn from feedback and adjust behavior accordingly declines with age (Marschner et al., 2005; Mell et al., 2005; Eppinger et al., 2011; Hämmerer et al., 2011; Ferdinand, 2019). However, the neural mechanisms underlying age-related chances in choice behavior during learning remain unclear. The present study was aimed at elucidating the relation between learning-related neural processes and changes in behavior during probabilistic learning.

Processes that occur during learning, such as feedback evaluation, integration of feedback information, and updating of knowledge, seem to be affected by age [for a review see Ferdinand and Czernochowski (2018)]. For example, neural activity related to the difference between expected and actual feedback after an action or choice (reward prediction error) is less distinct in older compared to younger adults (Mata et al., 2011; Samanez-Larkin et al., 2014; Ferdinand and Czernochowski, 2018). However, these changes do not seem to be driven by reductions in the sensitivity to reward. For instance, studies using functional magnetic resonance imaging (fMRI) have indicated that the same set of brain areas appear to be activated following positive and negative feedback in young and older adults, and the fronto-striatal response to positive feedback remains intact for older individuals (e.g., Schott et al., 2007; Cox et al., 2008; Samanez-Larkin et al., 2014). In sum, age-related learning differences do not seem to hinge on reward sensitivity, but rather on processes during feedback evaluation and knowledge updating.

Medial prefrontal cortical brain areas have been shown to play a pivotal role in learning-related processes, and the medial prefrontal anterior cingulate cortices (ACC) are thought to be especially important, as they have been shown to be active during the evaluation and integration of feedback information (Alexander and Brown, 2011; Cohen et al., 2011; Samanez-Larkin et al., 2014; Kolling et al., 2016). Furthermore, ACC activity during learning has been shown to depend on age, with older adults displaying less distinct neural patterns in these brain regions compared to young adults when evaluating feedback (Eppinger et al., 2008; Hämmerer et al., 2011; Samanez-Larkin et al., 2014).

The fact that the neural processes involved in feedback evaluation change with age can have implications for the ability to learn from feedback. For instance, behaviorally, older adults have been shown to develop a preference for processing either positive or negative feedback, depending on task demands (Eppinger et al., 2011). In a task where feedback does not have to be used for learning, for example when stimulus-reward associations are known in advance, older adults seem to focus less on negative feedback compared to young adults, as illustrated by reduced neural activity in older adults when anticipating negative but not positive feedback. In contrast, if successful performance in the future depends on feedback processing (e.g., in probabilistic learning tasks), negative feedback has a stronger impact on learning-related choice behavior of older adults compared to positive feedback. Ferdinand (2019), for example, showed that reducing the information value of negative feedback hindered older adults' learning performance more than reducing the information value of positive feedback, supporting the view that older adults rely more the negative feedback to learn in a probabilistic learning task.

Following the evaluation of feedback information, that information then needs to be used to update knowledge so it can potentially be used to guide the adjustment of subsequent behavior. Processes of knowledge updating have been linked to neural activity in fronto-parietal brain areas (Borst and Anderson, 2013). In addition, EEG studies have found neural evidence for increased knowledge updating prior to behavioral adaptation in the form of switching to a different response on the next trial (Polich, 2007; Chase et al., 2011; San Martin et al., 2013; Correa et al., 2018). The impact of age on this switch-choice behavior has been only sparsely studied, but the available studies show that both young and older adults adapt their switch choice on the next trial based on the feedback information received (Frank and Kong, 2008; Hämmerer et al., 2011). In addition, older adults displayed a bias toward negative feedback in that they modified their behavior to a lesser extent following positive feedback, a bias not found in young adults, further supporting the view that learning from positive feedback is diminished with age in probabilistic learning tasks (Frank and Kong, 2008; Hämmerer et al., 2011). Importantly, although older adults have been shown to evaluate feedback differently and adapt their choice behavior differently following feedback, the neural underpinnings of these effects remain unknown.

In order to gain more insight into the neural basis of age-related differences in feedback evaluation and knowledge updating during feedback learning, a group of younger and older participants performed a probabilistic learning task during which functional MRI measures of their brain activity were collected. The probabilistic learning task, which was based on a previous paradigm developed by our group (van den Berg et al., 2019), entailed participants choosing between houses and faces on each trial, after which they received either positive or negative feedback that was later converted into money. To gain money, participants had to actively learn which out of two stimulus types was more likely to yield a positive feedback for each of the 10 blocks of 20 trials.

In our study, older adults were expected to perform worse on the learning task compared to younger adults, and this diminished performance was expected to be paralleled by decreased neural activity related to feedback evaluation following positive feedback. In addition, we expected effects related to knowledge updating, which we investigated by contrasting neural activity related to switch behavior on the next trial. Given that we expected diminished knowledge updating in older adults, we predicted a smaller difference in neural activity related to making a switch vs. not making a switch on the next trial, in the older adult group compared to young adults. Furthermore, these differences in feedback evaluation and knowledge updating were expected to result in different choice behavior in older adults related to learning performance. If decreased learning performance in older adults is related to different feedback evaluation and knowledge updating, as we expected, the difference in choice behavior between the age groups would be most prominent following positive feedback. In contrast, if only knowledge updating is affected, we expected the difference in choice behavior to not depend on the valence of the feedback received.

## Materials and Methods

### Participants

There were 29 young and 27 older adults in this study. Participants were recruited by means of local advertisements and advertisements on social media. All participants were right-handed, had normal or correct-to-normal vision, and did not report taking any psychoactive medications. The study was conducted according to protocols approved by the Medical Ethical Committee of the University Medical Center Groningen, and all participants gave prior written informed consent. Four participants did not perform the task according to task instructions (three younger and one older adult, all female) and were therefore excluded from the final analysis. In addition, two older participants (one female) were excluded due to technical problems. Accordingly, the data from 26 young [13 female; 18–27 years; mean age (SD) = 22.2 (2.7)] and 24 older [11 female; 60–73 years; mean age (SD) = 65.5 (4.0)] participants were included in the analysis. Participants received 16 euros in compensation for their time, plus reimbursement of travel expenses and a variable monetary reward depending on their task performance [mean (SD): 5.50euro (4.40)].

### Experimental Tasks and Stimuli

#### Materials

MRI data were acquired on a Siemens 3T scanner at the University Medical Center Groningen. The probabilistic learning task was programmed using the Presentation® software package (Neurobehavioral Systems, Inc., Berkeley, CA, www.neurobs.com) and presented on an MRI-compatible IPS LCD monitor (BOLDscreen 24, Cambridge Research Systems, resolution 1920 × 1200) that was made visible to the participant through a mirror (viewing distance ~100 cm). Responses were made using two buttons positioned above each other on an MRI-compatible response box. The stimulus base set of 20 male face images and 20 house images (135 × 180 pixels) was identical to the one used by our group in van den Berg et al. (2019).

#### Probabilistic Learning Task

**Figure 1:**
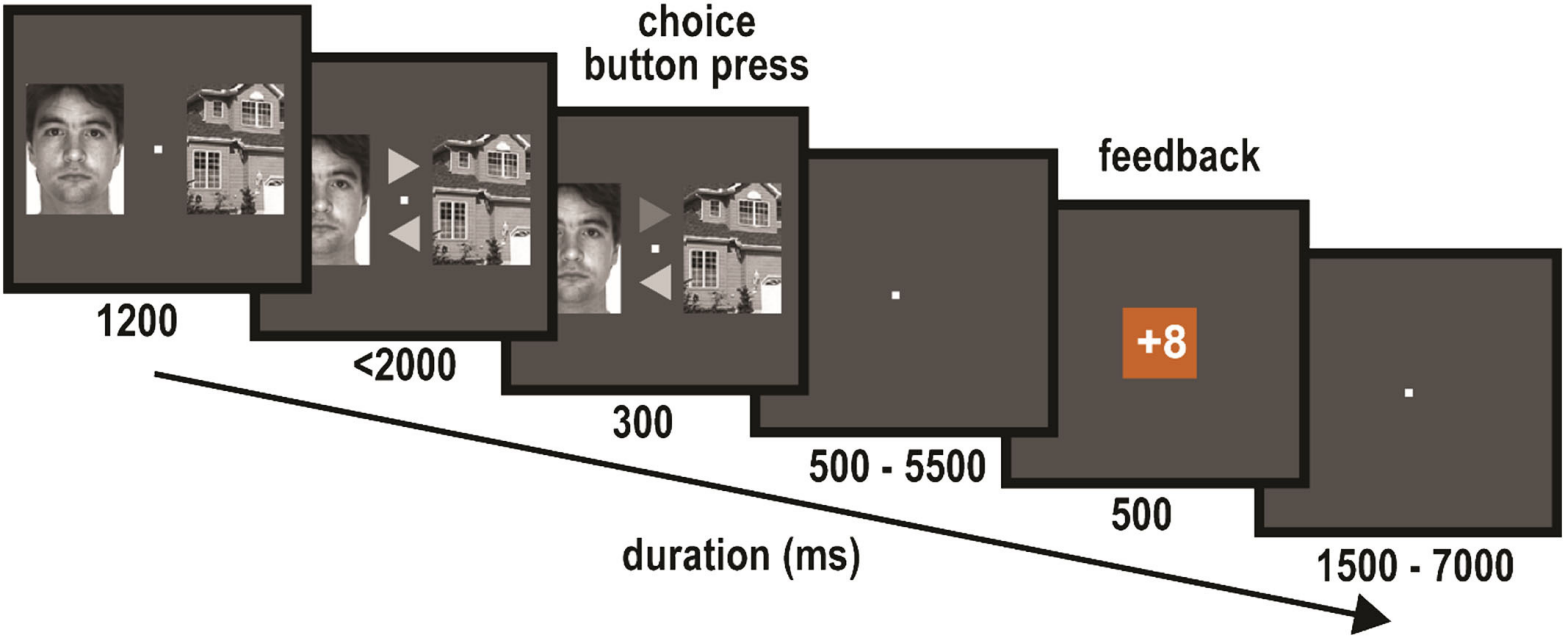
Probabilistic learning task: on each trial an image pair of a house and a face were shown (left/right location randomized over trials), with the task to choose one or the other. After the arrow stimuli appeared, participants indicated their choice by selecting the appropriate arrow. Following a jittered interval, feedback as to whether they won or loss was presented. Each experimental block consisted of 20 of these trials, during which participants learned which of the two image types was more likely to lead to a gain in that block. The human image in this figure is one of the stimuli we used in our experiment, which is part of an image-dataset that is publicly available and free for academic use. The source of the image-dataset is: http://www2.ece.ohio-state.edu/~aleix/ARdatabase.html (Martinez and Benavente, 1998).

Participants performed a probabilistic learning task (Figure 1) in which they were asked to maximize their gains by learning which of two stimulus types (houses or faces) was more likely to yield a gain. The task was very similar to the task used in the EEG study of van den Berg et al. (2019), with slight adaptations of the stimulus presentation and response times. The task consisted of 10 blocks of 20 trials each. In each 20-trial block, choosing either the face images or the house images was more likely to lead to a gain (62.5 vs. 37.5%). This stimulus type will be called the “block winner.” Each trial started with a pair of images, one of a face and one of a house, with one image presented to the left and the other to the right of a central fixation (Figure 1). The stimuli for this image-pair choice stimulus were randomly drawn from the base set (see materials section) and randomly assigned to be presented to the left and right locations. After 1,200 ms, two arrows appeared just above and below the fixation, each pointing to one of the two stimuli on the left and the right. The mapping of the arrow locations (above and below) to the direction the arrows pointed was randomly assigned on each trial. Participants indicated their choice using the response box by selecting the arrow pointing to the image they thought was more likely to yield a gain in that block. The selected arrow was highlighted for 300 ms, which was followed by a jittered interval (500–5,500 ms) during which a blank screen with fixation was shown. Following this interval, the feedback for that trial appeared on the screen for 500 ms. Feedback could either be a loss of eight points or a gain of eight points, presented as “−8” or “+8” printed in an orange or blue square (100 × 100 pixels) for a duration of 500 ms. The combination of square color and gain/loss was counterbalanced over participants. If participants did not respond within 2,000 ms following the presentation of the image pair at the beginning of the trial, the text “No response” appeared on the screen, which was then followed by a loss of eight points. The feedback stimulus was followed by a jittered presentation (1,500 – 7,000 ms) of a blank screen with a fixation cross, before the next image-pair choice stimulus appeared. Participants were instructed to try to maximize their gains by learning which of the two images was more likely to lead to a gain on the trials of a block. They were also informed about the probabilities of winning after choosing the block-winner (62.5%) vs. winning after choosing the block-loser (37.5%). In addition, participants were informed before the experiment started that their total score would be converted into a monetary reward at the end of the study. After each block, a short summary was shown that indicated the points that had been earned in that block and the total number of points accumulated up to that point in the session.

### Procedure

Prior to the experimental session, participants filled out the Apathy Evaluation Scale (Marin et al., 1991; Reijnders et al., 2010), as varying degrees of apathy can affect reward processing, especially in older adults. None of the participants scored above the cut-off for clinically relevant apathetic symptoms (cut-off = 37/38; Max. AES-score_young_ = 22; Max. AES-score_old_ = 25), and the average apathy scores did not differ between the two age groups [average AES-score_young_ = 16.1 (SD = 3.5); average AES-score_old_ = 16.0 (SD = 4.6); *t*_(41.1)_ = 0.1, *p* = 0.92]. Before entering the MRI scanner, participants received task instructions and practiced one block of the probabilistic learning task on a laptop. MRI acquisition started with a T1-weighted anatomical image, after which the participants performed a short classification task (which is not relevant for this paper), followed by two sessions of the probabilistic learning task, each consisting of five blocks. A short break was given between the two five-block sessions.

### Behavioral Analysis

Behavioral analyses were performed using R (R Core Team, 2020). A mixed modeling approach was used to model two types of learning behavior (choosing the block winner and switching between stimulus categories) as a function of both trial position (1–20) and age group. All models included a random intercept per participant to account for individual variation in average learning performance and switch rate. A second-degree polynomial of trial number was added if it improved the model fit significantly to account for non-linear associations (which was the case for the learning rate model). A random slope for trial number per participant was found to improve the model fit of all models significantly and was therefore added to account for individual differences in learning rate and switch behavior. In addition, we also modeled switch behavior based on feedback history (effect of feedback on trial_n−1_ and trial_n−2_ on switch choice behavior on trial_n_), to assess the impact of feedback integration effects on switch behavior. Again, a random intercept per participant was added to account for individual variation in average feedback integration effects, and random slopes for feedback (trial_n−1_ and trial_n−2_) per participant were considered to account for individual differences in the impact of each feedback position. Lastly, in an exploratory analysis, switch behavior was modeled based on a combination of feedback (trial_n−1_), trial position in the block (1–20), age group, and learning rate, to examine whether the impact of feedback on switch behavior changed across the 20-trial block. In addition to the random intercept per participant to account for individual variation in switch behavior, two random slopes were added to account for individual differences in the impact of feedback on switch behavior and the impact of trial position on switch behavior.

Models were estimated using the lme4 and AFEX package in R (Bates et al., 2015; Singmann et al., 2020), using likelihood ratio tests for fitting. Model comparison was performed using the Akaike information criterion (AIC) (Akaike, 1973), with a delta AIC-threshold of 2 (lower value indicating a better model fit). Data was pulled over all blocks, and statistical tests were considered significant at *p* < 0.05. Less than one percent of the data consisted of no responses [mean (SD): 0.9% (1.1%); min: 0% max: 5%]; these trials were not included in the analysis. No-response trials, trials preceded by a no-response trial, and the first trial of each block were excluded from the switch analyses, as these could not be classified as a switch or no-switch trial.

### MRI Acquisition

A high resolution T1-weighted anatomical image was collected using a 64-channel head coil. Functional data were acquired with an echo-planar imaging (EPI) sequence (TR = 1.25 s, TE = 30 ms, 60 axial slices, 2.0 × 2.0 × 3.0 mm voxel size).

### MRI Analysis

#### Preprocessing

All MRI analyses were performed using SPM12 toolbox (http://www.fil.ion.ucl.ac.uk/spm/). Before preprocessing the data were checked for excessive movements that could not be corrected for. No participants were excluded from further analysis. Functional data were corrected for slice acquisition time, and realignment was applied to correct for head movements. Then the images were registered to the corresponding anatomical image, and all images were spatially normalized to the ICBM space template. The data were spatially smoothed using a 6 mm Gaussian kernel.

#### General Linear Model Analysis

Three general linear models (GLM's) were fitted on the individual subject data to examine different learning-related processes (feedback evaluation, feedback integration, and knowledge updating). For the feedback evaluation model, gain trials and loss trials were added to the design matrix as predictors of interest. On the individual subject level, beta-estimates were calculated for gains (ß_gain_) and losses (ß_loss_) that were subsequently used to compare the two age groups using second-level contrasts (e.g., Young ß_gain_ – ß_loss_ – Old ß_gain_ – ß_loss_).

For the feedback integration model, four predictors were constructed based on the possible feedback combinations on the previous and current trial (e.g., gain-loss, loss-loss, etc.). The individual beta estimates of the different feedback sequences were used in second-level analyses to assess the effects of integration across trials. More specifically, we examined whether neural activation following feedback presentation of a loss or gain depended on whether it was preceded by a gain or loss on the previous trial. For example, neural differences identified when comparing gain-loss vs. loss-loss would be indicative of previous-trial feedback (gain vs. loss) impacting the processing of current loss feedback, which would imply feedback integration over trials. Similarly, we compared neural activity following a gain on the current trial depending on whether it was preceded by a gain or a loss (loss-gain vs. gain-gain). These effects were subsequently compared between the age groups [e.g., Young (ß_gain−loss_ − ß_loss−loss_) − Old (ß_gain−loss_ − ß_loss−loss_) and Young (ß_loss−gain_ − ß_gain−gain_) − Old (ß_loss−gain_ − ß_gain−gain_)].

Finally, to examine brain activation related to knowledge updating, two predictors reflecting switch and no-switch choice behavior on the next trial were constructed and added to the GLM model. The switch predictor contained all trials where the participants would switch their choice between stimulus types (i.e., choose face vs. choose house) on the next trial. The no-switch predictor consisted of all trials where the participant would choose the same stimulus type on the next trial. On the individual subject level, beta-estimates were calculated for switch trials (ß_switch_) and no-switch trials (ß_noswitch_), which were subsequently used in second-level analyses to assess group differences.

All models contained six movement covariates to account for head movements during the task, and all regressors were convolved with a canonical hemodynamic response function (HRF). The GLM analyses modeled the BOLD activity for a period of 2,000 ms starting at the moment of feedback presentation, based on previous EEG-work showing that the processes of feedback evaluation and knowledge updating last approximately this long (van den Berg et al., 2019). A high-pass filter with a 128-s time constant was used to remove low frequency drifts. All second-level contrasts were evaluated by whole-brain voxel-wise *t*-tests with a *p*-value threshold of 0.001 (uncorrected) and a minimal cluster-size of five voxels. Furthermore, FWE-corrections at the cluster level were applied (*p*_FWE_ < 0.05).

## Results

### Behavioral Results

#### Learning Rate

**Figure 2:**
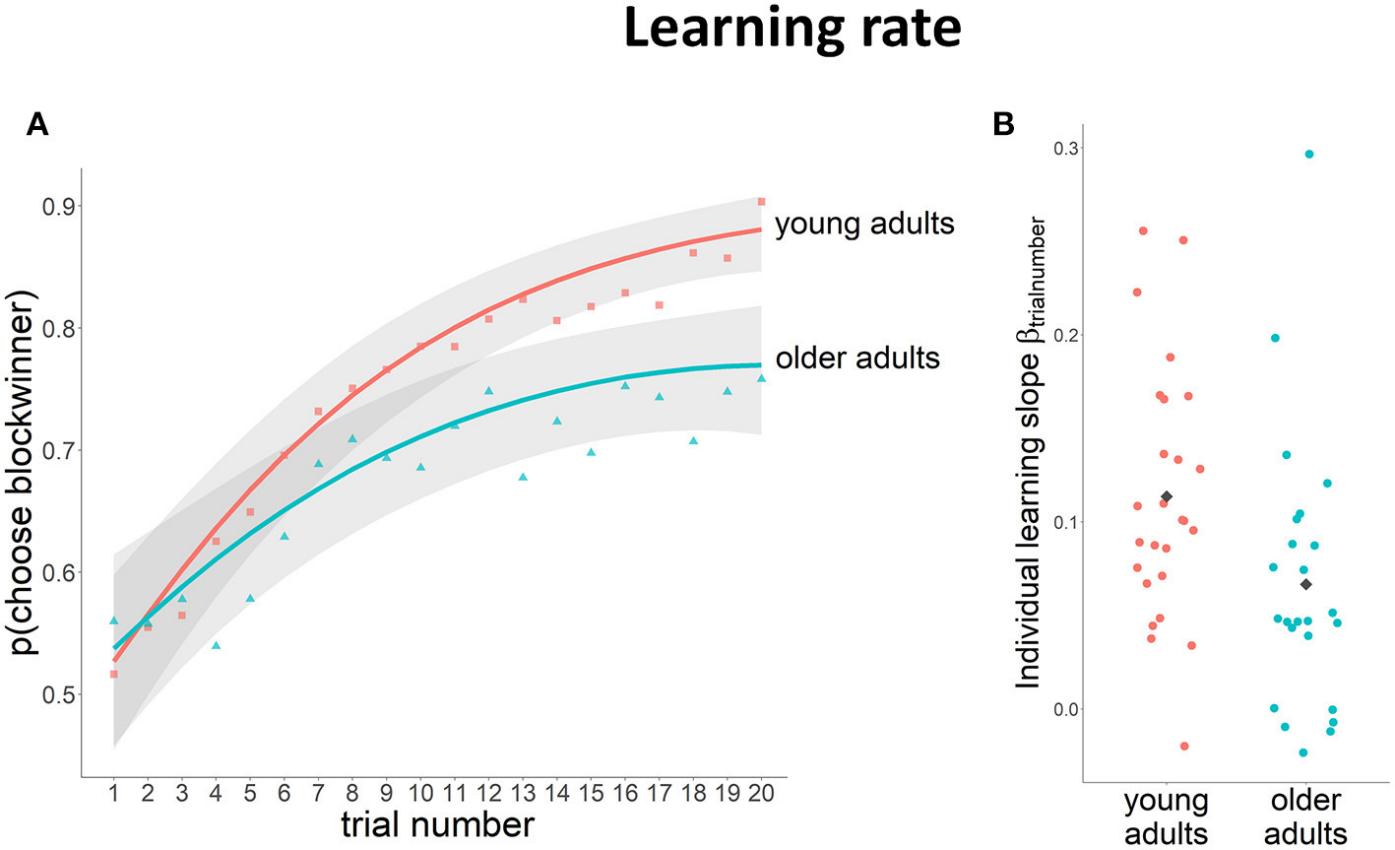
Learning rate data (lines depict the logistic model fit with a 95% CI in gray, raw data represented as separate points). **(A)** Probability of choosing the block winner on the different trials across the block for young and older adults; **(B)** estimated individual learning rates, gray diamonds represent the average learning rate per group.

Analyses of task performance showed that the probability of choosing the block winner increased across the block for both young and older participants, indicating the ability to learn which stimulus was more likely to yield a gain within the block [χ^2^(2) = 151.75, *p* < 0.01] (Figure 2A). *Post-hoc* tests revealed that, at the start of the block (i.e., on the 1st trial), both younger and older participants chose the block winner around chance level (younger adults: *p*_(chooseblockwinner)_ = 0.52 vs. older adults: *p*_(chooseblockwinner)_ = 0.53; odds ratio = 0.06, SE = 0.21, n.s.). At the end of the block (i.e., on the 20th trial), the probability of choosing the block winner was higher in both age groups, but was higher in younger participants compared to older participants [*p*_(chooseblockwinner)_ = 0.88 vs. *p*_(chooseblockwinner)_ = 0.77, respectively; odds ratio = 0.80, SE = 2.06, *p* < 0.01], reflecting a higher learning level for the younger group.

On average, younger participants had a steeper learning curve than older adults [χ^2^(2) = 16.78, *p* < 0.01; average learning rate_young_ = 0.11 (SD = 0.07); average learning rate_old_ = 0.07 (SD = 0.07)]. However, we also observed large individual differences in learning rate (Figure 2B). Hence, to further investigate if differences in brain activity in subsequent analysis of the fMRI BOLD signal can be explained by general age-related differences or by differences in learning rate, we took into consideration learning rate as a potential confounding factor in these analyses.

#### Switch Behavior

**Figure 3:**
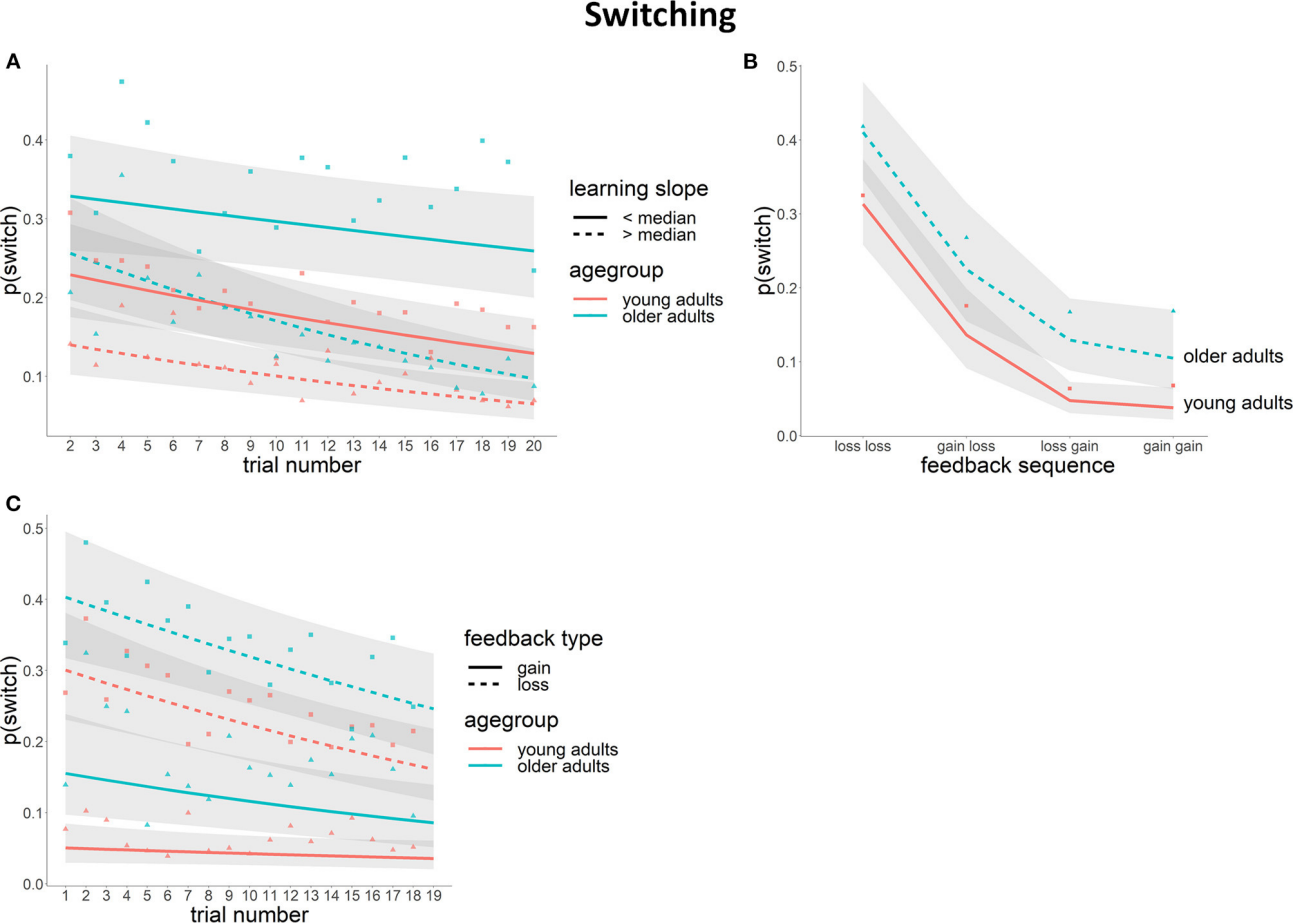
Switch behavior [lines depict the logistic model fit with a 95% CI in gray, raw data (indicating the proportion of switch) are depicted as separate points]. **(A)** switch probability across the block for young and older participants with high and low learning rates (median split per age group); **(B)** switch probability per feedback sequence for young and older adults; **(C)** switch probability across the block per feedback type and age group.

Switch probability (i.e., choosing a different stimulus category [houses or faces] on the current trial than on the previous trial) decreased over the course of a 20-trial block [χ^2^(1) = 24.16, *p* < 0.01]. Although on average the older group switched more often than younger participants [mean number of switches (SD): young = 29 (19), old = 46 (26); χ^2^(1) = 8.52, *p* < 0.01], the decrease in switch probability over the block was not significantly different between the two age groups [χ^2^(1) = 0.18, n.s.]. When taking into account the individual differences in learning performance in the mixed model, we found that higher learning rates were associated with a lower average switch probability [χ^2^(1) = 12.40, *p* < 0.01]. These effects were found to be dependent on an interaction between age and trial number [Age × Trial number × Learning rate: χ^2^(1) = 6.52, *p* = 0.01]. *Post-hoc* testing showed that switch probability during the block was similar for young adults with low learning rates and older adults with high learning rates. Young adults with high learning rates had a lower switch probability compared to the other groups and older adults with low learning rates had significantly higher switch probabilities compared to the other three groups (Figure 3A).

#### Effects of Feedback Information

In a further analysis we examined whether switch behavior was modulated by feedback history. More specifically, we inspected the impact of feedback valence (gain or loss) on the probability to make a switch by taking into account in the model both feedback on the previous trial (feedback_n−1_) and feedback on the trial before that (feedback_n−2_). Switch probability was higher following a loss compared to a gain [Feedback_n−1_: χ^2^(1) = 110.68, *p* < 0.01]. In addition, feedback on the trial two trials back also influenced the switch probability [Feedback_n−2_: χ^2^(1) = 42.68, *p* < 0.01]; however, this effect was dependent on feedback_n−1_ [Feedback_n−1_ × Feedback_n−2_: χ^2^(1) = 16.57, *p* < 0.01]. As reflected through *post-hoc* tests, the probability to make a switch was highest if both feedback_n−1_ and feedback_n−2_ were losses, and lowest after receiving two gains or a loss followed by a gain [*p*(switch)_loss−loss_ = 0.36, *p*(switch)_gain−loss_ = 0.18, *p*(switch)_loss−gain_ = 0.08, *p*(switch)_gain−gain_ = 0.06]. Feedback history effects were found not to differ between the two age groups in the model without the individual learning slopes [Feedback_n−2_ × Feedback_n−1_ × Age: χ^2^(1) = 0.02, n.s.] (Figure 3B), but the higher switch rate in older compared to young adults was more pronounced following a gain as compared to a loss [Age × Feedback_n−1_, χ^2^(1) = 4.59, *p* = 0.03]. After adding individual learning slopes to the model, the age effects were no longer significant [main effect of Age: χ^2^(1) = 2.28, n.s.; Age × Feedback_n−1_ interaction: χ^2^(1) = 2.98, n.s.]. Instead, learning rate was predictive of the switch probability, with higher learning rates being related to a lower overall switch probability [χ^2^(1) = 5.73, *p* = 0.02]. After accounting for individual learning slopes, no interactions between feedback history and age or learning rate remained.

Lastly, in addition to our planned analyses we examined whether the relation between feedback and switch probability changed over the course of the 20-trial block. An interaction was found [Feedback_n−1_ × Trial position: χ^2^(1) = 3.85, *p* = 0.05], indicating that whereas the switch probability following a gain remained the same over the course of the block, switch probability following a loss decreased over the block (Figure 3C). This effect was found not to differ with age [Feedback_n−1_ × Trial position × Age: χ^2^(1) = 2.95, n.s.] or learning rate [Feedback_n−1_ × Trial position × Age: χ^2^(1) = 1.36, n.s.]. Hence, overall we found that older adults switched more compared to young adults, but we found no evidence for different learning behavior between the two groups.

### fMRI Results

#### Feedback Evaluation

**Figure 4:**
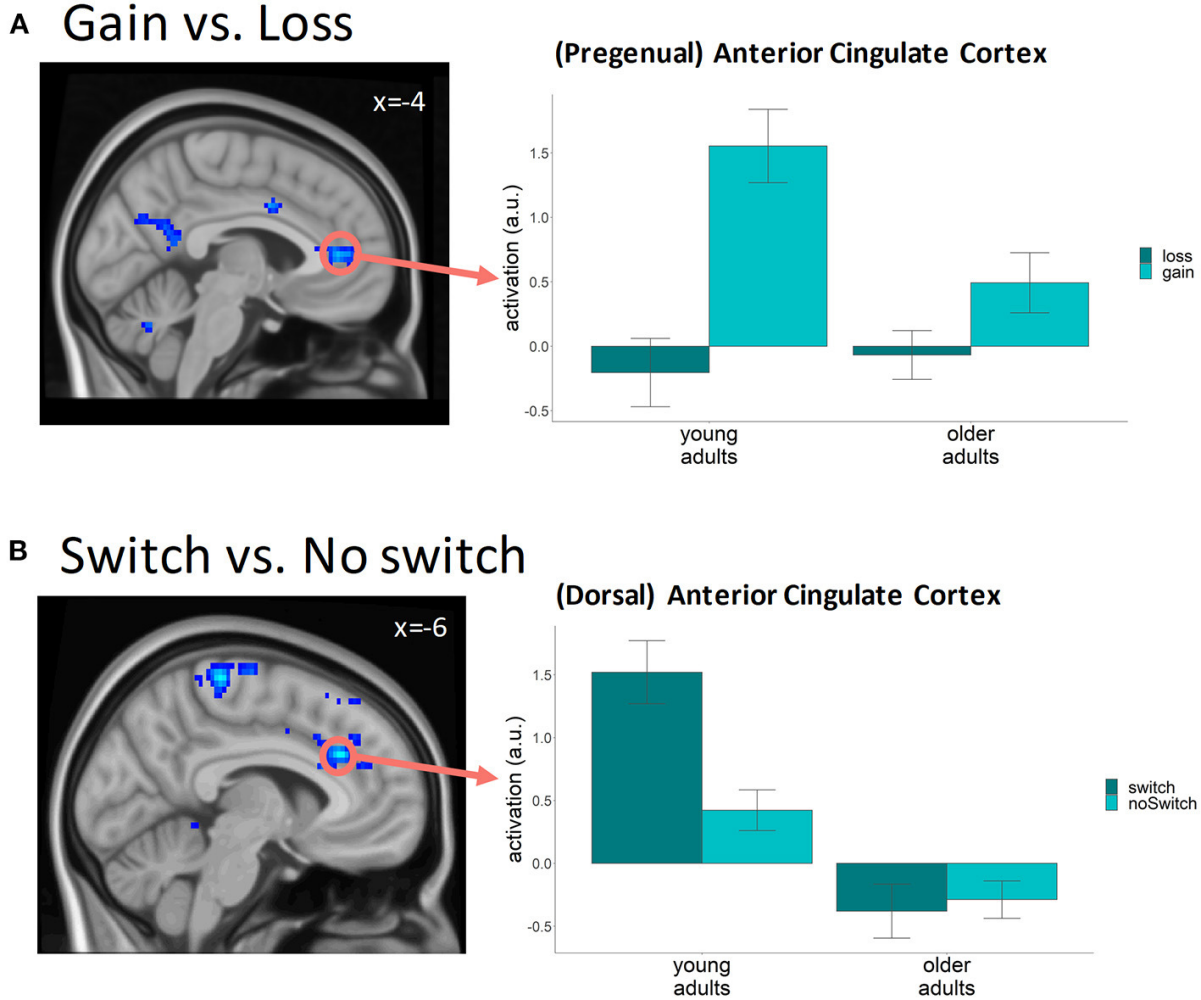
fMRI results (*p* < 0.001 uncorrected voxel level, *p*_FWE−cluster_ < 0.05) **(A)** Cluster covering the anterior cingulate cortex where the difference in brain activity between gains and losses was bigger in young compared to older participants. Bar graph was based on a 5 mm sphere ROI centered around the approximate center of the ACC cluster (MNI −4, 40, 8); **(B)** Clusters where the difference in BOLD activity preceding a switch on the next trial compared to a no-switch is bigger in young as compared to older participants. Bar graph is based on a 5 mm sphere ROI centered around the peak voxel in the left anterior cingulate gyrus (MNI −6, 28, 29).

We found clusters with higher levels of BOLD activity following gains compared to losses throughout the brain, in frontal, parietal, temporal, occipital, and subcortical regions, in line with previous research (e.g., Cohen et al., 2008; Drueke et al., 2015; Andreou et al., 2017) (See Supplementary Table S1 for a full overview of clusters). Conversely, we also found a trend toward higher activity following a loss compared to a gain in a small part of the frontal operculum, a part of the superior frontal gyrus, and in a part of the supplementary motor area and middle and anterior cingulate cortex (more superior compared to the gain activity effect), areas previously reported as being more active following negative feedback (Cohen et al., 2008; Bischoff-Grethe et al., 2009; Amiez et al., 2016). In addition to the general effects of feedback valence, we also found differences in feedback-related BOLD activity between the two age groups (contrast: young_gain>loss_ > old_gain>loss_), especially in parts of the middle frontal gyrus, anterior cingulate cortex, and angular gyrus. The involvement of these areas in feedback evaluation (Cox et al., 2005; Cohen et al., 2008; Mies et al., 2011; Jahn et al., 2014; Lee and Kim, 2014; Drueke et al., 2015) and the diminished increase in activity following gains in older compared to younger adults observed here (Figure 4A), suggest that especially the processing of positive feedback is reduced with age. Although previous studies have reported a stronger ventral striatal response to positive feedback in older adults (Schott et al., 2007; Widmer et al., 2017), we did not find an effect of age in this area. In addition to the age effects, our results showed that activity following feedback was also different for participants with a low versus a high learning rate. More specifically, the difference between gains and losses was more pronounced for the participants with a high learning rate in three frontal lobe clusters covering areas related to executive functioning, such as the supplementary motor cortex, middle cingulate gyrus and the postcentral gyrus.

#### Switch-Related Activity

In order to examine knowledge-updating effects, we compared levels of BOLD activity following feedback presentation on trials preceding a trial on which participants switched to the other stimulus category (switch trials), relative to trials preceding a no-switch trial. Of particular interest were the frontal and centro-pariatal brain areas, given that they are thought to be the origin of the LPC component linked to switch behavior by EEG research (Chase et al., 2011; San Martin et al., 2013). In the visualization (Figure 4B) we focused on the anterior cingulate cortex given its role in the processing of feedback and the updating of beliefs (Shenhav et al., 2013).

We found higher levels of activity preceding switch trials in frontal and parietal brain areas, the cerebellum, and the insular cortices (for a full overview of significant clusters see Supplementary Table S2). A direct comparison between the two age groups showed that switch-specific activity was more pronounced in young compared to older participants in three clusters, one covering parts of the anterior and middle cingulate cortex, the superior frontal gyrus, and the supplementary motor cortex, one covering parts of the precentral and postcentral gyrus, and one covering parts of the middle and superior frontal gyrus (Figure 4B). These results suggest enhanced knowledge updating in young adults prior to making a switch. Switch-related enhancement of activity was not observed in the older group, suggesting a less pronounced link between knowledge updating and choice behavior in this group. Besides the age-related effects, the increase in activity preceding switch compared to no-switch trials was found to be more pronounced in the group with high learning rates compared to low learning rates in parts of the middle frontal gyrus, medial superior frontal gyrus, and anterior cingulate cortex related to executive functioning.

#### Feedback History

The integration of feedback information over trials was examined by comparing neural activity following different feedback sequences to investigate if activity following current feedback depended on feedback on the previous trial (e.g., comparing a gain-loss sequence to a loss-loss one). Our results showed that neural activity after receiving gains and losses depended on the feedback received on the previous trial. When a gain was preceded by a loss as compared to a gain, more activity was found in areas related to reward processing, including the superior frontal gyrus, the supramarginal gyrus, and the anterior insula, suggesting that receiving negative feedback enhances reward processing of positive feedback on the next trial (see Supplementary Table S3 for full results). No significant clusters were found where activity was higher when the gain was preceded by a gain as compared to when it was preceded by a loss. With regard to receiving negative feedback, we found enhanced activity in the inferior temporal gyrus and fusiform gyrus when a loss was preceded by a gain compared to a loss. When a loss was preceded by another loss we found higher levels of activity in areas involved in feedback evaluation and decision making, including like the medial superior frontal gyrus and anterior insula, suggesting that receiving negative feedback enhances processing of the subsequent loss.

Taken together, these results suggest that feedback processing is impacted by preceding feedback, and especially by negative feedback. Direct group comparisons did not reveal any significant differences in the effect of feedback history between young and older participants. In participants with a high learning rate compared to a low learning rate, however, we did find a larger enhancement of activity in the right anterior insula and frontal operculum when a loss was preceded by another loss compared to a gain, suggesting a more prominent effect of previous feedback in participants with high learning rates when receiving negative feedback.

## Discussion

In this study, we aimed to elucidate age-related differences in the neural mechanisms underlying learning. Older and younger adults performed a probabilistic learning task in which they were asked to learn which one out of two stimulus types was more likely to lead to a gain. The behavioral results indicate that over the course of a block of 20 trials, participants increasingly chose the option that was more likely to yield a gain, showing that they were able to learn from the feedback. However, in line with previous research, we also found that older participants had more difficulty learning from the probabilistic feedback compared to young participants, as indicated by a lower learning rate (Mell et al., 2005; Hämmerer et al., 2011). This diminished performance in older adults coincided with altered choice behavior, as well as in differences in feedback evaluation and knowledge updating at the neural level.

In order to gain more insight in how differences in learning performance arise, it is valuable to examine the choice behavior leading up to this performance in more detail. During our task, participants were given feedback on each trial, and based on this information they could decide whether or not to switch their choice from one stimulus to the other on the next trial. Our results show that participants indeed used feedback information to adapt their choice behavior, switching more often following a loss compared to a gain. However, because of the probabilistic nature of the feedback, participants knew to expect a loss occasionally even when choosing the correct stimulus (i.e., block winner). Therefore, feedback information should not be used only at a trial level, but it should also be integrated over trials in order to learn which of the two choice options was more likely to be the block winner and to choose that option more and more often. In line with this, the probability to switch to the other stimulus was found to not only depend on the most recent feedback, but also on feedback history. Furthermore, switch behavior was shown to be related to learning performance, with the making of fewer switches in general being associated with a higher learning rate. Together these results indicate that switch choice behavior was guided by feedback information and linked to learning performance, underlining its relevance in the study of feedback related learning.

As feedback information was accumulated over the course of each 20-trial block, participants were expected to become more certain about which stimulus was the block winner and, as a result, become more consistent in their choices. This was reflected in the decreasing number of switches across the block, in combination with the increasing chance of choosing the block winner. At the same time, this growing bias toward choosing the block winner across the block can be expected to coincide with requiring more conflicting evidence before switching to the other stimulus. In other words, because of the probabilistic feedback, participants will be more likely to accept more losses when they are more convinced their choice is right. As certainty about the identity of the block winner is higher at the end of the block compared to the beginning, participants are expected to accept more losses at the end of the block before adapting their choice behavior. We indeed showed that switch probability following a loss decreased across the block, indicating that the information value of the feedback decreased as participants became more certain of the block winner. These results show how choice behavior was not only adapted based on feedback information received, but also as participants learned the right choice (e.g., with time on task).

Importantly, switch choice behavior and its modulation depended on age. First of all, older adults were found to switch more in general compared to younger adults, regardless of feedback valence or trial number within the block. One of the factors that might be related to this difference in switch tendency is the uncertainty of probabilistic feedback. It could be argued that older adults are less able to use relative uncertainty to guide learning (Nassar et al., 2016). Given that the value of feedback at the start of the 20-trial block is more uncertain compared to the end of the block when the participant has learned the stimulus reward associations, optimal use of uncertainty in the learning process would implicate a decreasing switch tendency following negative feedback across the block associated with the declining level of uncertainty. Suboptimal use of uncertainty in older adults would therefore imply a reduced adaptation of switch tendency across the block, which is not in line with our findings of a comparable modulation of the probability that participants shift between the choice options across the block in the two age groups following negative feedback, suggesting that the higher switch rate in older adults is most likely not driven by a less efficient use of uncertainty during learning. However, the higher general switch rate in older adults in combination with the fact that this higher tendency was more pronounced following positive compared to negative feedback, might also suggest that older adults adhere to a different learning strategy that results in alternative choice behavior and less successful learning. Our findings are in line with findings of Hämmerer et al. (2011), who also reported a larger age-related difference in switch rate following positive feedback. They argued that this suggests that switch choice behavior is affected less strongly by positive feedback in older compared to younger adults, meaning that that older adults learn less from positive feedback compared to negative feedback. Taken together, our results suggest that age impacts learning-related behavior at the trial level, both with regard to the magnitude of switch choice tendency and how it is affected by feedback valence.

In line with the fact that the influence of positive feedback on subsequent choice behavior was different between the two age groups, our fMRI analysis showed age-related differences related to the processing of positive feedback. More specifically, we found that even though neural activity levels following gains were higher compared to losses in both age groups, older adults demonstrated a less differential BOLD response to gains and losses in brain areas that have been associated with, amongst other things, feedback evaluation (e.g., ACC, Alexander and Brown, 2011; Neubert et al., 2015; Kolling et al., 2016; Bradley et al., 2017; Wiseman et al., 2018). ROI visualizations indicate that this smaller differential response to gains and losses was due to a diminished neural response to positive feedback (i.e., because BOLD activity increased less compared to baseline following positive feedback in the older adults, the activity levels following gains and losses were more similar in this group). A less differential neural response to feedback valence in older adults has also been reported in EEG-studies in which smaller differences in the feedback related negativity (FRN) component were found (Mathewson et al., 2008; Hämmerer et al., 2011), which has been argued to reflect a reduced ability to discriminate feedback valence in light of the task goal (Hämmerer et al., 2011). Our results show that age effects were specifically driven by a smaller increase in activity in brain areas following positive feedback in older compared to younger adults. In combination with our behavioral results, this suggests that positive feedback is processed differently in the older group, potentially altering its contribution to the learning process, and resulting in a diminished ability to make optimal behavioral changes. We did not replicate previous findings of a stronger ventral striatal response to the rewarding feedback in older as compared to younger adults (Schott et al., 2007; Widmer et al., 2017), which might be related to differences in task characteristics between our study and previous research. In the probabilistic learning task that we used, participants had to continuously learn which stimulus type was associated with a higher chance of winning, while the stimulus-reward associations in these previous studies were known beforehand, thereby reducing the degree of uncertainty in a task. This difference might have an impact on the information value of feedback information and the influences of feedback on subsequent behavior.

When comparing neural activity related to the different feedback sequences, higher levels of activity were found in the inferior temporal gyrus and fusiform gyrus when negative feedback was preceded by positive versus negative feedback. As these brain areas have been associated with the processing of faces and houses, it could be that these results reflect cortical (re)activation of stimulus processing areas as part of establishing, updating and storage of stimulus-reward associations, as we reported earlier in van den Berg et al. (2019). However, our dataset does not lend itself for a more detailed analysis of this effect, and thus this interpretation remains speculative and would need to be confirmed by future research.

In addition to feedback evaluation, we examined the neural processes related to switch choice behavior in order to investigate potential age-related differences in knowledge updating. Previous EEG-research showed that making a switch on the next trial was accompanied by a larger Late Positive Complex (LPC) component from 300 to 600 ms post feedback stimulus, which was thought to reflect enhanced brain activity in frontal and centro-parietal brain areas prior to a switch (Fischer and Ullsperger, 2013; San Martin et al., 2013). One of the potentially contributing brain areas of particular interest is the anterior cingulate cortex (ACC), which is thought to be serving as a central hub in the allocation of cognitive control and is involved in processing of feedback and updating of beliefs (Shenhav et al., 2013).

In line with the EEG-studies (Chase et al., 2011; San Martin et al., 2013; Correa et al., 2018), our results indeed showed increased activity in the ACC following feedback presentation on the trial prior to a switch in young adults, which might reflect increased levels of knowledge updating prior to behavioral adjustment. In older adults, however, no evidence was found for increased ACC activity on the trial prior to a switch, suggesting that updating of knowledge was less effective in this group. Based on the results of the present study, we cannot be certain whether the equivalent levels of switch- and no-switch related neural activity in older adults specifically reflected relatively diminished knowledge updating prior to a behavioral switch or relatively enhanced knowledge updating prior to not switching in comparison to young adults. In addition, as switch choice behavior is inherently linked to feedback valence (higher switch tendencies following negative compared to positive feedback), and fMRI has limited temporal resolution, distinguishing between feedback evaluation and knowledge updating processes is limited with this method. The low temporal resolution of fMRI does not allow us to temporally distinguish processes related to feedback evaluation feedback integration and knowledge updating, thus restricting us to interpreting our results based on the specific contrasts showing the differences in activation. This however does not guarantee that the activation patterns are exclusive for these processes. Nevertheless, the distinct spatial locations of the reported feedback-evaluation and knowledge-updating effects, corresponding to diminished brain activity in feedback-evaluation areas following positive feedback in older adults, and a less prominent increase of activity in areas involved in the updating of beliefs prior to adaptations in choice behavior in this group, seem to indicate that age impacts both feedback processing and knowledge updating during learning.

In conclusion, successful learning requires the evaluation of feedback, updating of knowledge, and adaptation of behavior. We provided evidence showing that diminished learning performance in older adults corresponded with making more switch choices. In addition, older adults were shown to process positive feedback to a lesser extend compared to young adults, which might have led to the reduced knowledge updating we found in this group. Together our findings showed how learning-related processes are impacted by age both at the behavioral and neural levels, and how different feedback-evaluation processes could lead to reduced learning performance across trials.

## Data Availability Statement

The datasets generated for this study are available on reasonable request. Requests to access the datasets should be directed to Berry van den Berg, berry.van.den.berg@rug.nl.

## Ethics Statement

The studies involving human participants were reviewed and approved by The University Medical Center Groningen medical ethics committee. The participants provided their written informed consent to participate in this study.

## Author Contributions

BB, MW, and ML: study design. TH and BB: data acquisition and data processing. TH, BB, MW, AA, and ML: manuscript writing. All authors contributed to the article and approved the submitted version.

## Conflict of Interest

The authors declare that the research was conducted in the absence of any commercial or financial relationships that could be construed as a potential conflict of interest.
