# Supplementary material for: Diminished Feedback Evaluation and Knowledge Updating Underlying Age-Related Differences in Choice Behavior During Feedback Learning
Source: Front Hum Neurosci. 2021 Mar 5;15:635996. doi: 10.3389/fnhum.2021.635996 (PMC7973460; doi:10.3389/fnhum.2021.635996)
Supplement: Supplementary file 1 [file Data_Sheet_1.pdf]

Supplementary Material

*S1. fMRI results Gain vs. Loss analysis. For each cluster the area of the peak voxel is listed first, followed by the other areas in the cluster. Minimal cluster size=5, p<.001 uncorrected voxel level and FWE cluster correction p<0.05 (0.05>p_{fwe}<0.10 also reported to provide insight into trends). L/R= left and/or right hemisphere, * marks parts of the same cluster.*

Cluster areas	***L/R***	***MNI coordinates peak voxel***	***Cluster size***	***$p_{fwe-corr}$ cluster***
GAIN>LOSS				
Frontal				
*[Superior frontal gyrus, Middle frontal gyrus, Anterior cingulate gyrus, Inferior frontal gyrus (triangular part), Superior frontal gyrus (medial segment), Central operculum, Middle cingulate gyrus, Precentral gyrus (medial segment)]**	L/R	-	-	-
Lateral orbital gyrus	L	-38,50,-10	87	0.077
[*Middle frontal gyrus, Anterior orbital gyrus]*				
Parietal				
*[Angular gyrus, Precuneus, Posterior cingulate gyrus, Supramarginal gyrus, Parietal operculum]**	L/R	-	-	-
Temporal				
*[Middle temporal gyrus, Superior temporal gyrus, Planum temporale, Planum polare]**	L/R	-	-	-
Middle temporal gyrus	L	-58,-48,-7	195	0.002
[Inferior temporal gyrus]				
Occipital				
*[Middle occipital gyrus]**	L/R	-	-	-
Cerebellar				
Cerebellum exterior	L/R	44,-72,-34	2231	<0.001
[Cerebellar vermal lobules VI-VII, Cerebellar vermal lobules VIII-X]				
Insula	L	-	-	-
*[Posterior insula, anterior insula]**				
Subcortical				
Putamen	L/R	22,12,-7	13478	<0.001

Cluster areas	***L/R***	***MNI coordinates peak voxel***	***Cluster size***	***$p_{fwe\text{-}corr}$ cluster***
*[Putamen, Caudate, Hippocampus]**				
YOUNG$_{GAIN>LOSS}$ > OLD$_{GAIN>LOSS}$				
Frontal				
Middle frontal gyrus	L	-26,12,50	203	0.002
[Superior frontal gyrus]				
Anterior cingulate gyrus	L	-26,44,8	126	0.020
Parietal				
Angular gyrus	L	-42,-66,29	146	0.011
HIGH LEARNING RATE$_{GAIN>LOSS}$ > LOW LEARNING RATE $_{GAIN>LOSS}$				
Frontal				
Supplementary motor cortex	L/R	6,-2,56	759	<0.001
[Middle cingulate gyrus, Precentral gyrus (medial segment), Superior frontal gyrus]				
Precentral gyrus	L	-28,-12,59	681	<0.001
[Postcentral gyrus, Superior frontal gyrus]				
Precentral gyrus	R	30,-12,59	260	<0.001
[Postcentral gyrus, Superior parietal lobe, Superior frontal gyrus]				

S2. fMRI results Switch vs. No Switch analysis. For each cluster the area of the peak voxel is listed first, followed by the other areas in the cluster. Minimal cluster size=5, p<.001 uncorrected voxel level and FWE cluster correction p<0.05 (0.05>p_{fwe}<0.10 also reported to provide insight into trends). L/R= left and/or right hemisphere.

**Cluster areas**	**L/R**	**MNI coordinates peak voxel**	**Cluster size**	***$p_{fwe-corr}$ cluster***
SWITCH > NO SWITCH				
**Frontal**				
Superior frontal gyrus	L/R	14,12,62	1313	<0.001
[Superior frontal gyrus (medial segment), Supplementary motor cortex, Anterior cingulate gyrus, Middle cingulate gyrus]				
Middle frontal gyrus	R	32,56,20	450	<0.001
[Superior frontal gyrus]				
Middle frontal gyrus	L	-32,52,17	216	0.002
[Superior frontal gyrus]				
Parietal				
Angular gyrus	R	54,-54,35	365	<0.001
[Supramarginal gyrus]				
Cerebellar				
Cerebellum Exterior	L	-30,-62,-28	107	0.047
Insula				
Frontal operculum	R	44,16,-1	480	<0.001
[Anterior insula, inferior frontal gyrus (opercular part and orbital part), posterior orbital gyrus]				
Anterior insula	L	-28,22,-4	215	0.002
[Frontal operculum, Posterior orbital gyrus]				
YOUNG$_{SWITCH>NO\ SWITCH}$ > OLD$_{SWITCH>NO\ SWITCH}$				
Frontal				
Anterior cingulate gyrus	L/R	-6,28,29	1184	<0.001
[Superior frontal gyrus (medial segment), Middle cingulate gyrus, Supplementary motor cortex]				
Precentral gyrus (medial segment)	L/R	2,-30,68	424	<0.001

**Cluster areas**	**L/R**	**MNI coordinates peak voxel**	**Cluster size**	***$p_{fwe\text{-}corr}$ cluster***
[Postcentral gyrus, Precentral gyrus, Postcentral gyrus (medial segment)]				
Middle frontal gyrus	R	28,54,20	206	0.003
[Superior frontal gyrus]				
HIGH LEARNING RATE$_{SWITCH>NO\ SWITCH}$ > LOW LEARNING RATE$_{SWITCH>NO\ SWITCH}$				
Frontal				
Middle frontal gyrus, Superior frontal gyrus (medial segment), anterior cingulate gyrus	L	-14,42,20	102	0.056

S3. fMRI results Feedback integration analysis. For each cluster the area of the peak voxel is listed first, followed by the other areas in the cluster. Minimal cluster size=5, p<.001 uncorrected voxel level and FWE cluster correction p<0.05 (0.05>p_{fwe}<0.10 also reported to provide insight into trends). L/R= left and/or right hemisphere.

Cluster areas	L/R	MNI coordinates peak voxel	Cluster size	$p_{fwe\text{-}corr}$ *cluster*
**GAIN LOSS > LOSS LOSS**				
Temporal				
Fusiform gyrus	L	-44,-38,-13	218	0.001
[Inferior temporal gyrus, Middle temporal gyrus]				
Inferior temporal gyrus	R	46,-48,-13	176	0.005
[Fusiform gyrus]				
**LOSS LOSS > GAIN LOSS**				
Frontal				
Superior frontal gyrus (medial segment)	R	6,36,32	180	0.004
[Supplementary motor cortex]				
Inferior frontal gyrus (orbital part)	R	48,44,-10	121	0.025
[Inferior frontal gyrus (triangular part), Lateral orbital gyrus, Frontal operculum]				
Superior frontal gyrus	R	26,58,20	118	0.028
[Middle frontal gyrus]				
Middle frontal gyrus	R	42,16,53	90	0.073
Insula				
Anterior insula	L	-28,16,-10	114	0.032
[Frontal operculum, Posterior orbital gyrus]				
**LOSS GAIN > GAIN GAIN**				
Frontal				
Middle frontal gyrus	L/R	44,32,29	2348	<0.001
[Superior frontal gyrus, Supplementary motor cortex, Precentral gyrus, Superior frontal gyrus (medial segement), Inferior frontal gyrus (opercular part)]				

**Cluster areas**	**L/R**	**MNI coordinates peak voxel**	**Cluster size**	***p**$_{fwe-corr}$* ***cluster***
Superior frontal gyrus	L	-20,8,68	1941	<0.001
[Middle frontal gyrus, Precentral gyrus, Inferior frontal gyrus (opercular part and triangular part)]				
Parietal				
Supramarginal gyrus	R	46,-42,53	873	<0.001
[Angular gyrus, Superior parietal lobe]				
Supramarginal gyrus	L	-36,-46,41	852	<0.001
[Superior parietal lobe, Angular gyrus, Postcentral gyrus]				
Cerebellar				
Cerebellum exterior	L/R	-30,-62,-28	1753	<0.001
[Occipital fusiform gyrus, Cerebellar vermal lobules VI-VII]				
Insula				
Anterior insula	R	34,18,5	157	0.008
[Frontal operculum]				
Anterior insula	L	-34,18,-1	143	0.013
[Frontal operculum]				
Subcortical				
Caudate	R	14,0,17	88	0.078
HIGH LEARNING RATE**$_{LOSS\ LOSS>GAIN\ LOSS}$* ***> LOW LEARNING RATE *$_{LOSS\ LOSS>GAIN\ LOSS}$*				
Frontal				
Frontal operculum	R	42,16,2	137	0.015
[Anterior insula, Inferior frontal gyrus (orbital part)]				
